# Extraction outcomes of implantable cardioverter-defibrillator leads vary by manufacturer and model family

**DOI:** 10.1093/europace/euad345

**Published:** 2023-11-24

**Authors:** Katsuhide Hayashi, Thomas Callahan, John Rickard, Arwa Younis, Bryan Baranowski, David Martin, Shady Nakhla, Chadi Tabaja, Bruce L Wilkoff

**Affiliations:** Cardiac Electrophysiology and Pacing Section, Department of Cardiovascular Medicine, Cleveland Clinic, 9500 Euclid Avenue Desk J2-2, Cleveland, OH 44195, USA; Cardiac Electrophysiology and Pacing Section, Department of Cardiovascular Medicine, Cleveland Clinic, 9500 Euclid Avenue Desk J2-2, Cleveland, OH 44195, USA; Cardiac Electrophysiology and Pacing Section, Department of Cardiovascular Medicine, Cleveland Clinic, 9500 Euclid Avenue Desk J2-2, Cleveland, OH 44195, USA; Cardiac Electrophysiology and Pacing Section, Department of Cardiovascular Medicine, Cleveland Clinic, 9500 Euclid Avenue Desk J2-2, Cleveland, OH 44195, USA; Cardiac Electrophysiology and Pacing Section, Department of Cardiovascular Medicine, Cleveland Clinic, 9500 Euclid Avenue Desk J2-2, Cleveland, OH 44195, USA; Cardiac Electrophysiology and Pacing Section, Department of Cardiovascular Medicine, Cleveland Clinic, 9500 Euclid Avenue Desk J2-2, Cleveland, OH 44195, USA; Cardiac Electrophysiology and Pacing Section, Department of Cardiovascular Medicine, Cleveland Clinic, 9500 Euclid Avenue Desk J2-2, Cleveland, OH 44195, USA; Cardiac Electrophysiology and Pacing Section, Department of Cardiovascular Medicine, Cleveland Clinic, 9500 Euclid Avenue Desk J2-2, Cleveland, OH 44195, USA; Cardiac Electrophysiology and Pacing Section, Department of Cardiovascular Medicine, Cleveland Clinic, 9500 Euclid Avenue Desk J2-2, Cleveland, OH 44195, USA

**Keywords:** Implantable cardioverter-defibrillator lead, Transvenous lead extraction, Incomplete removal, Complications, Lead family

## Abstract

**Aims:**

Transvenous lead extraction (TLE) of implantable cardioverter-defibrillator (ICD) leads is considered challenging. The structure of each ICD leads is variable between manufacturer and model families. The net impact of lead family on the safety and effectiveness of TLE is poorly characterized. We assessed the safety and efficacy of ICD TLE and the impact of manufacturer ICD model family on the outcomes.

**Methods and results:**

The study cohort included all consecutive patients with ICD who underwent TLE between 2013 and 2022 and are enrolled in the Cleveland Clinic Prospective TLE Registry. A total of 885 ICD leads (median implant duration 8 years) in 810 patients were included. Complete ICD TLE success was achieved in 97.2% of the leads (*n* = 860) and in 98.0% of the patients (*n* = 794). Major complications occurred in 22 patients (2.7%). Complete procedural success rate varied by manufacturer and lead family; Medtronic 98.9%, Abbott 95.9%, Boston Scientific 95.0%, Biotronik 91.2%, *P* = 0.03, and Linox family leads had the lowest, 89.7% *P* = 0.02. Multivariable predictors of incomplete ICD lead removal included ICD lead age > 10 years and Linox family lead. Multivariable predictors of major complications included ICD lead age > 15 years and longer lead extraction time, and predictors of all-cause mortality within 30 days included lead extraction for infection, end-stage renal disease, and higher New York Heart Association functional class.

**Conclusion:**

Complete and safe ICD lead removal rate by TLE is extremely high but varied by manufacturer and lead family. Linox family lead and >10 years lead age were independent predictors of incomplete lead removal.

## Graphical Abstract

**Graphical Abstract:**
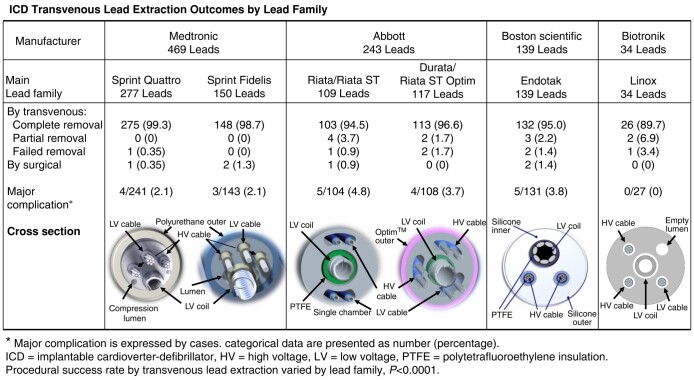
ICD transvenous lead extraction outcomes by lead family. *Major complication is expressed by cases. Categorical data are presented as number (percentage). ICD, implantable cardioverter-defibrillator; HV, high voltage; LV, low voltage; PTFE, polytetrafluoroethylene insulation. Procedural success rate by transvenous lead extraction varied by lead family, *P* < 0.0001.

**What’s new?**

- This study suggests that procedural success rate by implantable cardioverter-defibrillator transvenous lead extraction varies by manufacturer and lead family.
- Linox family lead and >10 years lead age were independent predictors of incomplete lead removal.

## Introduction

An ongoing challenge in the management of patients with implantable cardioverter-defibrillators (ICDs) is the increasing need to remove leads for lead dysfunction or infection.^1,2^ In addition, the clinical decision-making in choosing to extract or abandon no longer useful leads is now a frequent and difficult choice particularly with regard to maintaining an MR conditional cardiac implantable electronic device (CIED) system. Transvenous lead extraction (TLE) of ICD leads is considered challenging because fibrotic tissue growth and calcification are the main obstacle to lead removal, particularly after long dwell durations.^3^ Risk factors, such as dwell time, number of leads, lead fixation mechanism, and dual-coil lead design, have been identified as predictors of failure or complications related to the TLE procedure.

The structure of each ICD lead is complex, has multiple components, diverse materials and is distinct between manufacturer and lead model families. The distinctives of lead structure has been reported to influence TLE procedural success rate and complications.^4,5^ However, the net impact of lead family on the safety and effectiveness of ICD TLE is poorly characterized.

The goal of this study is to evaluate the safety and efficacy of ICD TLE and compare and assess the impact of manufacturer ICD model family on the safety and effectiveness of TLE in a large, prospectively collected, observational registry of TLE patients presenting for extraction of long implant duration ICD devices.

## Methods

### Patient population and data acquisition

The study cohort included all consecutive patients with ICD TLE in the Cleveland Clinic Prospective TLE Registry between February 2013 and April 2022. Lead extraction was defined as removal of a lead implanted for more than 1 year or requiring specialized extraction tools. Therefore, we excluded the patients who underwent TLE within 1 year of implantation by manual traction. All patients in which there was an attempt to extract at least one ICD lead were included. Demographic, historical, procedural data, and the date when death occurred after TLE were obtained from electronic medical records and prospectively collected institutional databases. The study was approved by the institutional review board of the Cleveland Clinic.

### Transvenous lead extraction procedure

The procedures were under general anaesthesia. Simple traction represents removal from the vascular space during gentle manipulation during the dissection of the lead and suture tie down sleeve in the pocket or with advancement of a standard stylet without the introduction of a locking stylet. If this gentle manipulation did not result in successful lead removal, each lead was prepared with a locking stylet and a firmly tightened suture tied on the insulation also extended and tied back to the locking stylet loop. Telescoping extraction sheaths, mechanical or powered (rotational or laser) sheaths, were advanced over the leads to release the leads from the fibrotic attachments. The extraction sheaths were either GlideLight Laser Sheath (Philips, Colorado Springs, CO), Evolution RL Mechanical Dilator Sheath (Cook Medical, Bloomington, IN), or TightRail (Philips). In some instances, mechanical sheaths, snares, and/or the femoral approach were needed to complete the extraction.

### Definition

#### Patient outcome

Patient outcomes of TLE were defined in accordance with the 2017 HRS consensus statement and 2018 European Heart Rhythm Association (EHRA) expert consensus statements.^6,7^

1. ‘Complete success' was classified as the removal of the entire lead system.
2. ‘Partial success' was defined as when most of the lead is removed, leaving <4 cm of coil and/or insulation and/or lead tip.
3. ‘Failure' was defined if more than ≥4 cm tip remained.

Patient outcome was classified according to the worst outcome of any of the leads.

#### Complication

Major complications were defined as those related to the procedure that were life threatening or resulted in death, or any unexpected event that caused persistent or significant disability, or any event that required significant surgical intervention. Minor complications were defined as requiring medical or minor procedural intervention.

#### Lead outcome

1. ‘Complete lead removal' was defined as the successful removal of all target lead material by transvenous tools only.
2. ‘Partial lead removal' was defined as when most of the lead is removed, leaving <4 cm of coil and/or insulation and/or lead tip.
3. ‘Failure' was defined if more than ≥4 cm tip remained.
4. ‘Conversion to surgical removal' was defined when conversion to surgical removal occurred with or without of a major complication.

Incomplete lead removal included ‘Partial lead removal', ‘Failure', and ‘Conversion to surgical removal'.

### Statistical analysis

Continuous variables are expressed as median (interquartile range, IQR: 25th–75th), whereas categorical variables are expressed as counts and percentages. Categorical variables were compared using χ^2^ test or Fisher’s exact test. Variables with *P* values of <0.1 after single variable analysis were entered into a multiple variable regression analysis in search of independent predictor of incomplete lead removal, major complication, or all-cause mortality within 30 days. *P* values of <0.05 was considered statistically significant. All statistical analysis was performed using JMP version 15.2.0 (SAS Institute Inc., Cary, NC).

## Results

### Patient’s characteristics, lead characteristics, and extraction methods in implantable cardioverter-defibrillator transvenous lead extraction

A total of 810 patients met inclusion criteria. Patient characteristics and the indications for extraction are listed in *Table 1*. The median patient age was 63 (52–71) years, and 577 (71.1%) were male. Infectious indications were present for almost 50% of the patients. Lead characteristics are listed in *Table 2*. Regarding manufacturer, Medtronic leads were most common (53%). Dual-coil ICD leads were used in approximately one-third of the patients. The median lead age was 8 (5–11) years.

**Table 1:** Patient characteristics (*n* = 810)

Demographics	
Age (years)	63.0 (52.0–71.0)
Gender: male, *n* (%)	577 (71.1)
Body mass index (kg/m^2^)	28.3 (24.8–33.1)
Medical history	
Coronary artery disease, *n* (%)	358 (44.2)
Cardiomyopathy, *n* (%)	630 (77.8)
Atrial fibrillation, *n* (%)	312 (38.5)
Hypertension, *n* (%)	471 (58.1)
Diabetes mellitus, *n* (%)	227 (28.0)
COPD, *n* (%)	86 (10.6)
ESRD on haemodialysis, *n* (%)	37 (4.6)
Left ventricular ejection fraction, %	41.0 (30.0–55.0)
New York Heart Association functional class Ⅲ or Ⅳ, *n* (%)	125 (15.4)
Prior open heart surgery, *n* (%)	248 (30.6)
Indication for lead extraction [*n* (%)]	
Infection	375 (46.3)
Lead malfunction	318 (39.3)
Recall lead	33 (4.1)
Upgrade of pre-existing system	25 (3.1)
No further indication	12 (1.5)
Externalization and recall lead	11 (1.4)
SVC syndrome	7 (0.9)
Abandoned lead	7 (0.9)
Breast cancer	5 (0.6)
Pain	4 (0.5)
Other	13 (1.6)

Continuous data are presented as median (interquartile range: 25th–75th); categorical data are presented as number (percentage).

COPD, chronic obstructive pulmonary disease; ESRD, end-stage renal disease; SVC, superior vena cava.

**Table 2:** Lead characteristics (*n* = 885)

	*n* = 885
Manufacturer	
Medtronic	469 (53.0)
Abbott	243 (27.5)
Boston Scientific	139 (15.7)
Biotronik	34 (3.8)
Coil	
Single coil	284 (67.9)
Dual coil	601 (32.1)
Fixation	
Passive	66 (7.5)
Active	809 (91.4)
Floating supra vena cava	10 (1.1)
Lead body size, mm	2.6 (2.2–2.8)
Lead age, year	8 (5–11)
Combined lead age, year	14 (6–26)
Number of lead removal per procedure	2 (1–3)
Lead extraction time, min	5 (3–10)

Continuous data are presented as median (interquartile range: 25th–75th); categorical data are presented as number (percentage).

**Figure 1:**
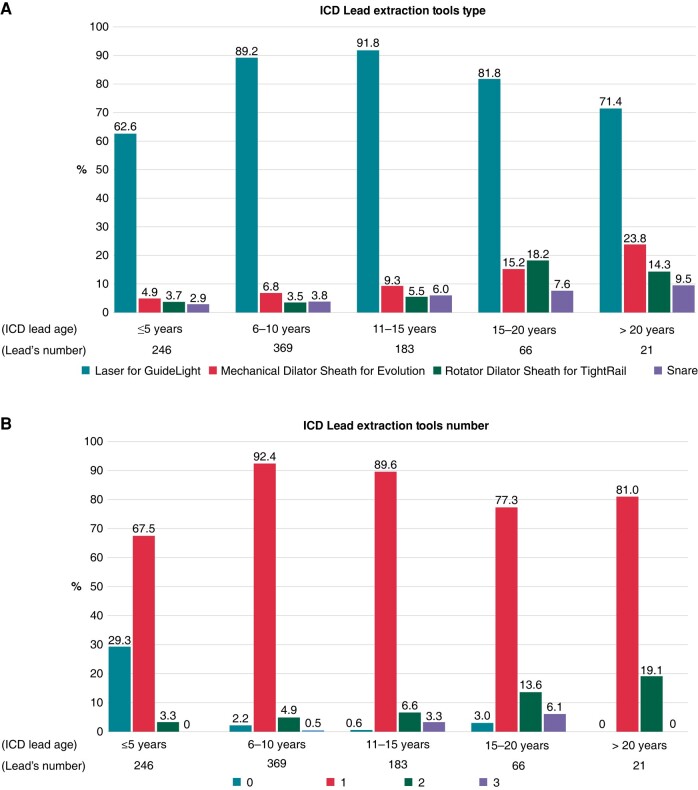
(*A*) Extraction tools type according to lead age. (*B*) Extraction tools number according to lead age. The required number of extraction tools during TLE broken down by ICD lead implant duration in 5-year increments. ICD, implantable cardioverter-defibrillator.

The extraction tools needed during TLE according to ICD lead age were illustrated in *Figure 1A*. The rate of cases requiring Laser for GuideLight, Mechanical Dilator Sheath for Evolution, Rotator Dilator Sheath for TightRail, or Snare for lead extraction tended to increase in proportion to the ICD lead age. However, in the cases with lead extraction of >15 years lead age, the use of Laser decreased and instead of the use of Evolution or TightRail increased. *Figure 1B* illustrates the required number of extraction tools during TLE broken down by ICD lead implant duration in 5-year increments. The number of required extraction tools increased with the extracted ICD lead age.

### Clinical outcome

The outcomes are summarized in *Table 3*. From the perspective of patient-level outcome, complete success was achieved in 794 of the 810 patients (98.0%), while procedural failure occurred in 9 patients (1.1%). Twenty-two patients (2.7%) had major complications and in 1 patient (0.1% incidence) periprocedural deaths occurred. From the perspective of lead-level outcome, 860 of total 885 leads (97.2%) were completely removed, 7 leads were partially removed, and 11 leads were failed removal. The remaining 7 leads were extracted surgically.

**Table 3:** Procedural outcomes

	810 patients
Patient-level outcome	
Clinical success	801 (98.9)
Complete success	794 (98.0)
Transvenous tools only	788 (97.3)
Transvenous plus surgical tools	6 (0.7)
Partial success	7 (0.9)
Failure	9 (1.1)
Complication	
Major complication	22 (2.7)
Intraoperative death	1 (0.1)
Minor complication	3 (0.4)
Lead-level outcome	885 leads
Transvenous lead removal	
Complete lead removal by transvenous tools only	860 (97.2)
Partial removal (<4 cm)	7 (0.8)
Failed removal	11 (1.2)
Conversion to surgical removal	7 (0.8)

Categorical data are presented as number (percentage).

**Figure 2:**
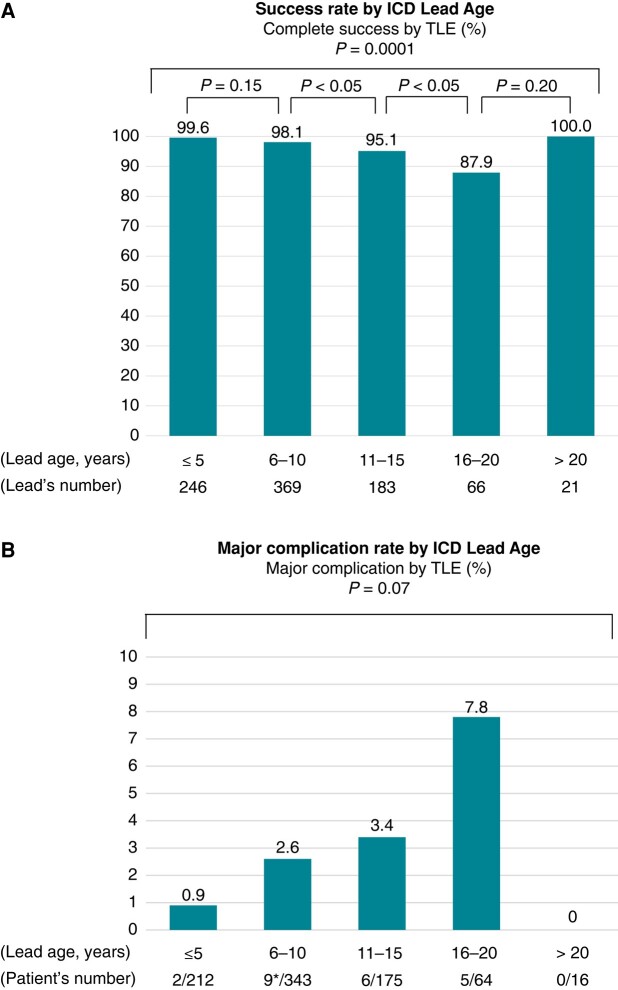
Outcomes by ICD lead age. (*A*) Complete success rate of TLE by ICD lead age. (*B*) Major complications by ICD lead age. ICD, implantable cardioverter-defibrillator; TLE, transvenous lead extraction.

Outcomes according to ICD lead age are demonstrated in *Figure 2*. Complete success rate of TLE differed significantly by ICD lead age, *P* = 0.0001. In the 5-year between-group comparison, complete success rate decreased significantly during 6–10 and 11–15 years, and during the 11–15 and 16–20 years ICD cohort leads. Major complications increased in leads 16–20 years of age, but not significantly, *P* = 0.07. There was one intraoperative death, which occurred in a patient with extraction of 9 years ICD lead.

In 20 of 22 cases with major complications, these complications were related to bleeding from vascular or cardiac chambers. The other two cases were caused by sepsis or flash pulmonary oedema, respectively. The locations of the injuries that occurred in cases requiring emergent surgical or endovascular intervention were summarized in *Table 4*. The superior vena cava (SVC) was most common site of injury in this group (10 of 20, 50%).

**Table 4:** Location of injury in cases requiring emergent surgical or endovascular intervention

	*n* = 20
Superior vena cava	10 (50)
Right ventricle	3 (15)
Superior vena cava-right atrial junction	2 (10)
Brachiocephalic vein	2 (10)
Left subclavian vein	2 (10)
Superior vena cava-brachiocephalic vein	1 (5)

Categorical data are presented as number (percentage).

All-cause mortality rate in 30 days was 3.5% (28 of 810 cases) in all patients. In patients with infectious indication, all-cause mortality in 30 days was 5.9% (22 of 375 cases) and leads were extracted 7 (4–10) days after diagnosis of device infection.

### Comparison of implantable cardioverter-defibrillator leads outcome by manufacturer and lead family

Procedural success rate varied by manufacturer, Biotronik leads had the lowest complete removal rate; (91.2%, *n* = 34 leads) vs. Medtronic (98.9%, *n* = 469 leads), vs. Abbott (95.9%, *n* = 243 leads), vs. Boston Scientific (95.0%, *n* = 139 leads), *P* = 0.003 (*Table 5*). Major complication rate did not differ between manufacturers. When analysed by lead family, Linox family leads had the lowest complete lead removal rate; (89.7%, *n* = 29) as compared to Sprint Quattro 99.3%, *n* = 277, Sprint Fidelis 98.7%, *n* = 150, Riata/Riata ST 94.5%, *n* = 109, Durata/Riata ST Optim 96.6%, *n* = 117, and Endotak 95.0%, *n* = 139, *P* = 0.02. Major complication rate did not differ between lead families. Implantable cardioverter-defibrillator lead age was youngest in Linox family leads, *P* < 0.0001. There were three incomplete removal cases in Linox family lead extraction. In two of three cases, we used a laser sheath first, but the lead fractured. A snare was then employed via femoral workstation and ultimately a <4 cm lead remnant remained. In another case of failed extraction by another hospital, we attempted extraction of the broken ICD lead by using a snare, but a >4 cm lead remnant remained, then we moved to surgical removal.

**Table 5:** Outcomes by manufacturer and lead family

Manufacturer	Medtronic 469 leads		Abbott 243 leads		Boston Scientific 139 leads	Biotronik 34 leads	*P* value
By transvenous complete removal	464/469 leads (98.9%)		233/243 leads (95.9%)		132/139 leads (95.0%)	31/34 leads (91.2%)	0.003
Major complication^a^	8/419 cases (1.9%)		9/228 case (4.0%)		5/131 cases (3.8%)	0/32 cases (0%)	0.28
Main lead family	Sprint Quattro 277 leads	Sprint Fidelis 150 leads	Riata/Riata ST 109 leads	Durata/Riata ST Optim 117 leads	Endotak 139 leads	Linox 29 leads	
By transvenous							0.02
Complete removal	275 (99.3)	148 (98.7)	103 (94.5)	113 (96.6)	132 (95.0)	26 (89.7)	
Partial removal	0 (0)	0 (0)	4 (3.7)	2 (1.7)	3 (2.2)	2 (6.9)	
Failed removal	1 (0.35)	0 (0)	1 (0.9)	2 (1.7)	2 (1.4)	1 (3.4)	
By surgical	1 (0.35)	2 (1.3)	1 (0.9)	0 (0)	2 (1.4)	0 (0)	
Major complication^a^	4/241 (1.7)	3/143 (2.1)	5/104 (4.8)	4/108 (3.7)	5/131 (3.8)	0/27 (0)	0.39
ICD lead age	6 (4–9)	10 (8–12)	10 (8–13)	6 (4–8)	9 (4–13)	6 (3–8)	<0.0001
Combined lead age	12 (6–24)	14 (10–24)	16 (9–27)	12 (6–24)	14 (8–28)	7 (6–11)	0.03
Dual-coil ICD leads	183 (66.1)	129 (86.0)	102 (93.6)	71 (60.7)	83 (59.7)	14 (48.3)	<0.0001
≥3 extraction tools	6 (2.2)	1 (0.7)	2 (1.8)	1 (0.9)	2 (1.4)	0 (0)	0.71
Extraction time, min	5 (3–10)	5 (3–10)	8 (4–15)	5 (3–15)	5 (3–10)	4 (2–13)	0.005

ICD, implantable cardioverter-defibrillator.

^a^Major complication is expressed by cases. Categorical data are presented as number (percentage).

### Predictor for incomplete implantable cardioverter-defibrillator lead removal and major complication

By multivariable analysis, ICD lead age > 10 years (OR 6.26, 95% CI 2.39–16.35; *P* = 0.0002) and Linox lead family leads (OR 11.30, 95% CI 2.41–53.06; *P* = 0.002) were independent predictors of incomplete lead removal (*Table 6*).

**Table 6:** Predictors for incomplete ICD lead removal

Incomplete ICD lead removal	Univariate analysis		Multivariable analysis	
	OR (95% CI)	*P* value	OR (95% CI)	*P* value
ICD leads age > 10 years	6.17 (2.55–14.95)	<0.0001	6.26 (2.39–16.35)	0.0002
Combined leads age, per 1 year	1.02 (0.99–1.04)	0.19		
Number of lead extracted	0.90 (0.60–1.34)	0.59		
Lead family				
Sprint Quattro	0.18 (0.04–0.79)	0.02	0.59 (0.12–2.96)	0.52
Sprint Fidelis	0.42 (0.10–1.80)	0.24		
Riata/Riata ST	2.32 (0.91–5.95)	0.08	2.66 (0.86–8.23)	0.09
Durata/Riata ST Optim	1.26 (0.43–3.74)	0.68		
Endotak	2.31 (0.95–5.65)	0.07	2.75 (0.93–8.14)	0.07
Linox	4.38 (1.23–15.56)	0.02	11.30 (2.41–53.06)	0.002
Coil				
Single coil	0.82 (0.34–1.99)	0.66		
Dual coil	1.22 (0.50–2.95)	0.66		
Fixation				
Passive	2.42 (0.81–7.28)	0.11		
Active	0.41 (0.14–1.24)	0.11		
Lead body size, per 1 mm	0.37 (0.09–1.63)	0.19		
Extraction for lead malfunction	1.96 (0.88–4.36)	0.10	1.53 (0.67–3.52)	0.31

CI, confidence interval; ICD, implantable cardioverter-defibrillator; OR, odds ratio.

Data from univariate and multivariate analyses for predictors of major complications are provided in *Table 7*. By multivariable analysis, ICD lead age > 15 years (OR 3.24, 95% CI 1.02–10.25; *P* = 0.046) and longer lead extraction time (OR 1.02, 95% CI 1.00–1.04; *P* = 0.01) were independent predictors of major complications. Difference of lead family was not associated with major complications.

**Table 7:** Predictors for major complications

Major complication	Univariate analysis		Multivariable analysis	
	OR (95% CI)	*P* value	OR (95% CI)	*P* value
Age, per year	0.98 (0.95–1.00)	0.07	0.98 (0.94–1.01)	0.22
Gender, female	2.11 (0.90–4.96)	0.09	2.18 (0.69–6.91)	0.19
ICD lead age > 15 years	2.95 (1.12–7.72)	0.03	3.24 (1.02–10.25)	0.046
Combined lead age, per 1 year	1.01 (0.99–1.04)	0.25		
≥3 leads extracted	0.75 (0.27–2.04)	0.56		
Dual-coil ICD lead	0.76 (0.32–1.84)	0.55		
Lead body size, per 1 mm	0.40 (0.08–1.94)	0.25		
Difference of lead family	—	0.97		
Required lead extraction tools	—	0.14		
Manual traction only				
1st tool				
2nd tool				
3rd or more tool				
Coronary artery disease	0.72 (0.30–1.72)	0.45		
ESRD on haemodialysis	0.99 (0.13–7.60)	0.99		
LV ejection fraction, per 1%	0.99 (0.97–1.02)	0.72		
NYHA class Ⅲ or Ⅳ	1.22 (0.41–3.68)	0.72		
SVC syndrome	—	0.53		
Lead extraction time, per 1 min	1.02 (1.01–1.04)	0.008	1.02 (1.00–1.04)	0.01

CI, confidence interval; COPD, chronic obstructive pulmonary disease; ESRD, end-stage renal disease; ICD, implantable cardioverter-defibrillator; NYHA, New York Heart Association; OR, odds ratio; SVC, superior vena cava.

### Predictor for all-cause mortality within 30 days

Data from univariate and multivariate analyses for predictors of all-cause mortality within 30 days from TLE are provided in *Table 8*. Multivariable analysis identified that lead extraction for infection (OR 2.66, 95% CI 1.00–7.10; *P* = 0.04), end-stage renal disease on haemodialysis (OR 3.54, 95% CI 1.13–11.10; *P* = 0.04), and New York Heart Association (NYHA) functional class Ⅲ or Ⅳ (OR 3.50, 95% CI 1.42–8.63; *P* = 0.007) were independent predictors.

**Table 8:** Predictors for all-cause mortality within 30 days

All-cause mortality within 30 days	Univariate analysis		Multivariable analysis	
	OR (95% CI)	*P* value	OR (95% CI)	*P* value
Age, per year	1.04 (1.01–1.07)	0.02	1.02 (0.99–1.05)	0.25
Gender, male	2.48 (0.85–7.24)	0.10		
Body mass index < 25 kg/m^2^	1.13 (0.49–2.61)	0.77		
ICD lead age, per 1 year	0.96 (0.88–1.04)	0.31		
Combined lead age, per 1 year	1.00 (0.98–1.03)	0.79		
Dual-coil ICD lead	1.63 (0.65–4.08)	0.29		
≥3 extraction tools use	2.86 (0.35–23.14)	0.32		
Lead extraction for infection	4.46 (1.79–11.11)	0.001	2.66 (1.00–7.10)	0.04
Difference of lead family	—	0.58		
Diabetes mellitus	1.70 (0.78–3.68)	0.18		
Coronary artery disease	1.48 (0.69–3.15)	0.31		
ESRD on haemodialysis	5.10 (1.82–14.27)	0.002	3.54 (1.13–11.10)	0.04
LV ejection fraction, per 1%	0.96 (0.93–0.99)	0.002	0.99 (0.96–1.02)	0.42
NYHA class Ⅲ or Ⅳ	5.18 (2.40–11.19)	<0.0001	3.50 (1.42–8.63)	0.007
Prior open heart surgery	2.34 (1.10–4.99)	0.03	1.47 (0.65–3.32)	0.35
Incomplete lead removal	2.93 (0.65–13.20)	0.16		

CI, confidence interval; ESRD, end-stage renal disease; ICD, implantable cardioverter-defibrillator; LV, left ventricular; NYHA, New York Heart Association; OR, odds ratio.

## Discussion

### Main findings

This study represents the largest reported study of ICD lead extraction and had the following main findings; first, complete ICD lead removal rate by TLE was extremely high, 97.2% with a median implant duration of 8.0 (5.0–11.0) years. Procedural outcomes varied by manufacturer or lead family, Biotronik and Linox family leads had the lowest complete lead removal rate, respectively. Lastly, multivariable predictors of incomplete ICD lead extraction included implant durations of >10 years and Linox family leads. Predictors for major complications were implant durations of >15 years and longer lead extraction time, and the predictors for all-cause mortality within 30 days were non-procedural, including extraction indication of infection, end-stage renal disease on haemodialysis, and higher NYHA functional class.

### Efficacy and safety for implantable cardioverter-defibrillator lead extraction by transvenous lead extraction

Recent reports of single-centre experiences of ICD TLE have reported high success rate.^3,8^ Ząbek *et al*.^8^ reported a success rate of 99.1% for 319 ICD lead extraction with 5.9 years lead age, and Segreti *et al*.^3^ reported a success rate of 99% for 678 ICD lead extraction with 4.0 years lead age. In our study, although implant duration was longer, overall of 8.0 years lead age, the procedure success rate of ICD lead extraction was extremely high, 98.9%. One of the reasons for this high success rate may be use of diverse lead extraction tools depending on the lead age as previously reported.^9^ ELECTRa and GALLERY registries showed that lead dwell time > 10 years was associated with clinical extraction failure.^10,11^ However, we were able to extract >20 years leads completely. We revealed that leads made by Medtronic showed the best procedural success rate compared with the other manufacturer’s ICD leads and the fact that Medtronic leads had the majority of >20 years ICD leads may have a positive impact on procedural success.

The incidence of major procedure-related complications of ICD lead extraction in this study was 2.7% (22 cases). This incidence is in line with previous reports. Ząbek *et al*.^8^ reported major complication rate of 2.2%, with a shorter implant duration 5.9 vs. 8.0 years. Furthermore, in our experience, injury occurred most often (65%) at SVC in cases requiring emergent surgical or endovascular intervention. This is concordant with our previously published experience with more than 5000 PM and ICD lead extractions.^12^ However, intraoperative death occurred only one case (0.1% incidence). The mortality was low compared with ELECTRa registry (0.5%). This mortality difference may be due to more healthy subjects in our study suggested by younger age (63.0 vs. 64.8 years in median age) and fewer patients with severe heart failure (15.4% vs. 32.0% in NYHA functional class Ⅲ or Ⅳ).

### The difference of implantable cardioverter-defibrillator lead construction and its relationship to extraction failure

**Figure 3:**
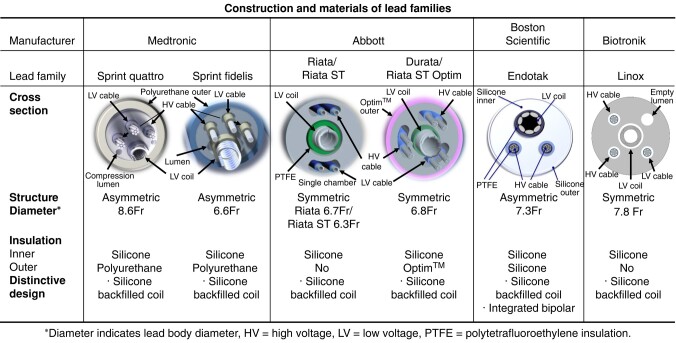
The difference of construction and materials by lead family.

We compared the outcomes of six ICD lead families from the four ICD lead manufacturers. In the present study, procedural outcomes differed between six lead families significantly. The Linox family lead was a multivariable predictor of incomplete or failed ICD lead removal, independent of lead age, number of extracted leads, percentage of dual-coil leads, and fixation mechanism. Constructions and materials of the six ICD lead families are summarized in *Figure 3*. Materials and structural design details are distinct between the six lead ICD lead families. Differences in lead construction include a more symmetric vs. asymmetric design. Linox lead and Riata/Riata ST lead have a ‘symmetric’ design. The difference in insulation also may matter. Silicone has poor resistance to tearing compared with polyurethane.^13^ To protect inner silicone insulation breach, Durata lead had an outer insulation coating with a copolymer consisting of silicone and polyurethane (Optim™), and Sprint Quattro and Sprint Fidelis lead have polyurethane outer insulation. While, Linox lead has silicone insulation without outer insulation. The lowest complete extraction rate of Linox lead may be due to these differences of construction and weaker tensile property compared with the other ICD lead families. In fact, both partial and failed lead removal rates were higher in Linox lead compared with the other lead families. Physicians should know the difference of lead properties by lead families before TLE.

It is important to keep in mind that there is discordance between the functional reliability of these ICD leads and ability to reliably extract these ICD leads. Two of the more reliably removed leads, Fidelis and Riata, were recalled for their electrical dysfunction rates. Both reliability and extractability are important and desirable.

### Clinical factors associated with major complication, all-cause mortality

Multivariable analysis showed that ICD lead age > 15 years was a predictor of major complications. Longer implant duration > 10 years was reported to associate with procedure-related major complication in ELECTRa registry for PM and ICD TLE.^10^ In our analysis for ICD TLE, it is noteworthy that major complication incidence increased in the case with >15 years ICD lead age. In addition to the ICD lead age, longer lead extraction procedural time predicted procedural complications. This is not surprising as fibrous adherence to the vessel and cardiac chambers due to fibrotic tissue growth and calcification must be overcome and the risk of complications are more a function the nature and strength of these attachments than the characteristics of the leads themselves. In fact, we showed that the difference by manufacturer or lead family was not associated with major complications. In previous studies, BMI < 25 kg/m^2^, female gender, longer implant duration, dual-coil ICD leads, lower LV ejection fraction, mechanical sheath, and powered sheath use have been associated with major complications during PM and ICD TLE.^10,12,14,15^ Notably, in our study for ICD TLE, BMI, gender, dual-coil ICD leads, and lower LV ejection fraction were not associated with major procedural complications.

Multivariable predictors of all-cause mortality within 30 days of ICD TLE were related to comorbid conditions, including lead extraction for infectious indications, end-stage renal disease, and NYHA functional class. These findings are consistent with the results of our previous analysis in more than 5000 PM and ICD lead extractions.^13^ There are no data/time overlap between our prior analysis.

### Study limitations

This was a retrospective study that may be affected by the possibility of unknown confounders and bias in management strategy, but the data were prospectively collected in consecutive patients. The data are derived from a single, high volume, tertiary care referral centre, and therefore may not be representative of care at the other centres. In addition, an unequal number of extracted ICD leads among lead family may limit the conclusion.

## Conclusions

We found that complete ICD lead removal rate by TLE was extremely high with a low incidence of major complication. Procedural success rate varied by manufacturer and lead family. Most importantly, regarding ICD TLE, multivariable predictors of clinical failure include implant duration of >10 years and Linox family leads. Predictors of major complications are implant durations of >15 years and longer lead extraction time. Finally, 30-day mortality is related to comorbidities but not related to procedural or lead characteristics.

## Data Availability

The data underlying this article will be shared on reasonable request to the corresponding authors.
